# Genetic signatures of small effective population sizes and demographic declines in an endangered rattlesnake, *Sistrurus catenatus*

**DOI:** 10.1111/eva.12731

**Published:** 2019-01-28

**Authors:** Michael Sovic, Anthony Fries, Scott A. Martin, H. Lisle Gibbs

**Affiliations:** ^1^ Department of Evolution, Ecology and Organismal Biology and Ohio Biodiversity Conservation Partnership The Ohio State University Columbus Ohio; ^2^ United States Air Force School of Aerospace Medicine Wright‐Patterson AFB Columbus Ohio; ^3^ Present address: College of Pharmacy The Ohio State University Columbus Ohio

**Keywords:** contemporary effective population size, eastern massasauga rattlesnake, forward genetic simulations, historical demographic modeling, microsatellites, RADSeq, *Sistrurus catenatus*

## Abstract

Endangered species that exist in small isolated populations are at elevated risk of losing adaptive variation due to genetic drift. Analyses that estimate short‐term effective population sizes, characterize historical demographic processes, and project the trajectory of genetic variation into the future are useful for predicting how levels of genetic diversity may change. Here, we use data from two independent types of genetic markers (single nucleotide polymorphisms [SNPs] and microsatellites) to evaluate genetic diversity in 17 populations spanning the geographic range of the endangered eastern massasauga rattlesnake (*Sistrurus catenatus*). First, we use SNP data to confirm previous reports that these populations exhibit high levels of genetic structure (overall Fst = 0.25). Second, we show that most populations have contemporary Ne estimates <50. Heterozygosity–fitness correlations in these populations provided no evidence for a genetic cost to living in small populations, though these tests may lack power. Third, model‐based demographic analyses of individual populations indicate that all have experienced declines, with the onset of many of these declines occurring over timescales consistent with anthropogenic impacts (<200 years). Finally, forward simulations of the expected loss of variation in relatively large (Ne = 50) and small (Ne = 10) populations indicate they will lose a substantial amount of their current standing neutral variation (63% and 99%, respectively) over the next 100 years. Our results argue that drift has a significant and increasing impact on levels of genetic variation in isolated populations of this snake, and efforts to assess and mitigate associated impacts on adaptive variation should be components of the management of this endangered reptile.

## 1 INTRODUCTION

Small, recently isolated populations face the risk of reduced viability over time due to genetic costs of inbreeding (Allendorf, Luikart, & Aitken, 2013). Habitat fragmentation can reduce gene flow between populations and decrease population size. This in turn increases the magnitude of genetic drift and the negative impact of inbreeding depression on survival and reproduction, and can lead to populations entering an “extinction vortex” (Gilpin & Soulé, 1986). Genetic analyses of historical and contemporary features of populations can provide information that is useful in assessing whether small isolated populations are at risk of high levels of drift and inbreeding depression, and thus should be significant components of conservation plans for endangered species (Jamieson & Allendorf, 2012).

One population feature that is important to understand in this context is effective population size (Ne) (Luikart, Ryman, Tallmon, Schwartz, & Allendorf, 2010), which measures the strength of genetic drift in a population. This is expected to reflect the rate at which variation is lost from populations and, in turn, may predict their ability to respond to future environmental change (Allendorf et al., 2013). Effective size is estimated over historical or contemporary timescales (Hare et al., 2011). Historical (long‐term) estimates of Ne evaluate the parameter over evolutionary timescales and generally estimate effective size at a species‐wide level (Charlesworth & Willis, 2009). In contrast, contemporary (current) measures of Ne estimate the parameter over short timescales (<5 generations) and so are most relevant to conservation and wildlife management applications (Hare et al., 2011).

The development of estimators of contemporary Ne using genetic data is an active area of research in conservation genetics (Waples, 2016; Waples, Larson, & Waples, 2016). Recently, Gilbert and Whitlock (2015) simulated data under a variety of demographic scenarios to evaluate different estimators of contemporary Ne and concluded that an estimator based on genome‐wide estimates of linkage disequilibrium (LDNe, Waples & Do, 2008), as implemented in the program NeEstimator (Do, Waples, & R.S., Peel, D., Macbeth, G.M., Tillett, B.J., and Ovenden, J.R., 2014) was accurate in small isolated populations. Likewise, Wang (2016) validated the use of a sibship‐based estimator implemented in the program Colony (Jones & Wang, 2010) under similar demographic scenarios. Both methods have been widely used for estimating contemporary Ne for captive and wild populations (Husemann, Zachos, Paxton, & Habel, 2016).

Analysis of observed distributions of genetic polymorphism can also yield insights into the demographic history of populations (Lawton‐Rauh, 2008; Marjoram & Tavare, 2006). To date, these analyses have primarily made qualitative inferences of changes in population size over time by comparing observed and expected patterns of diversity at small numbers of microsatellite loci (Garza & Williamson, 2001; Peery et al., 2012). However, these approaches can have low statistical power to detect even severe bottlenecks in natural populations (Busch, Waser, & DeWoody, 2007; Peery et al., 2012). Increasingly, alternative coalescent‐based modeling approaches are being used (i.e., Storz & Beaumont, 2002; Excoffier et al., 2013), which have the advantage of using an explicit statistical framework to test diverse demographic scenarios. These approaches have been used to detect significant demographic events over recent timescales, including those within the range of significant human impacts to the landscape (Goossens et al., 2006; Harris et al., 2016; Hoffman, Grant, Forcada, & Phillips, 2011). When combined with other information, the inferred timing of demographic events can be used to assess whether humans have played a significant role in those events (Goossens et al., 2006) or whether they are instead more likely due to natural large‐scale events, such as those associated with historical changes in climate (Tucker, Schwartz, Truex, Pilgrim, & Allendorf, 2012).

Finally, projecting future changes in genetic diversity can identify genetic risks faced by populations that have recently declined (Amos & Balmford, 2001). This issue is especially important when recent declines have led to populations not in genetic equilibrium. Populations with a small contemporary effective size may retain the genetic profile of a larger population after the decline, and this can lead to overestimates of the amount of variation present relative to present and future population sizes. Demographic simulations can help assess the rate at which existing levels of variation will decline and provide insights into an effective timeline for management activities such as genetic rescue (Frankham, 2015; Tallmon, Luikart, & Waples, 2004). One difficulty is that while the ultimate goal of management activities is the conservation of adaptive variation, these simulations are based on neutral evolutionary processes like genetic drift and focus on patterns of change in neutral variation (Carvajal‐Rodríguez, 2010). Nonetheless, they provide a time frame in which adaptive variation may be lost under the assumption that levels of adaptive and neutral variation covary (Reed & Frankham, 2003).

The eastern massasauga rattlesnake (*Sistrurus catenatus*) is a small short‐lived (generation time ~2 years) venomous snake found throughout eastern North America. In the United States, it primarily exists in relatively small, isolated patches of habitat surrounded by heavily modified landscapes (Szymanski et al., 2016). Population declines throughout the range due to habitat fragmentation and destruction have led to the listing of this species under the US Endangered Species Act (US Fish & Wildlife Service, 2016) in the United States and as a Species at Risk (Government of Canada, 2009) in Canada. This species exhibits little phylogeographic structure across its range (Sovic, Fries, & Gibbs, 2016), and so important management units within this species may best align with existing individual populations. Previous work at the population level (Chiucchi & Gibbs, 2010; Gibbs & Chiucchi, 2012) identified high levels of genetic structure among populations that have persisted over long periods of time, large variation in long‐term Ne between populations, and little evidence for recent declines in population sizes. However, these results were based on microsatellite‐based variation alone and used qualitative methods of analyses for inferring demographic patterns. In particular, measures of effective population size were historical long‐term measures. These may not reflect the strength at which drift is currently operating in these populations, making them uninformative for present‐day management decisions for this species.

Here, we use two independent genetic datasets to make conservation‐relevant inferences about genetic and demographic characteristics of individual populations across the range of this species. Our specific goals are to (a) evaluate the degree of genetic isolation among populations using a new genome‐scale dataset; (b) estimate contemporary effective population sizes to generate a better understanding of the present and future importance of drift in these populations; (c) re‐evaluate previous results (Gibbs & Chiucchi, 2012) that the genetic cost to living in small populations is small by using new estimates of individual heterozygosity from RADSeq loci in heterozygosity–fitness correlation (HFC) analyses (Chapman, Nakagawa, Coltman, Slate, & Sheldon, 2009); (d) test for evidence of past population size changes and estimate the timescale on which inferred changes occurred; and (e) use simulations to project the rate at which existing levels of variation will be lost in populations. Our study represents the most comprehensive analyses of the present‐day and future genetic demography of existing populations of *S. catenatus* and provides information useful for developing the Recovery Plan for this species (US Fish and Wildlife Service, in preparation).

## 2 METHODS

### 2.1 Samples

**Figure 1:**
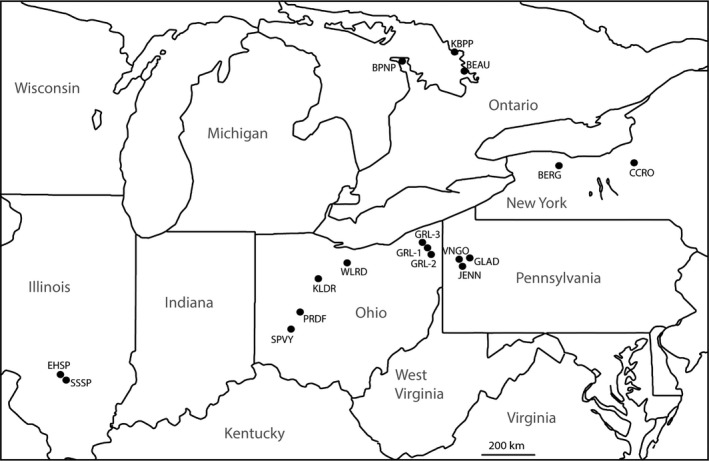
Map showing locations of populations of Sistrurus catenatus analyzed in this study. Summaries of genetic variation for RADSeq and microsatellite loci are given in Table 1

We analyzed genetic data from *S. catenatus* individuals from 17 populations spanning the geographic range of this species (Figure 1; Table 1). Individual populations were defined as sets of randomly collected adult samples from within a single geographic location ≤3 km^2^ in area for which sample sizes were sufficiently large to conduct the analyses described below (see Table 1 for marker‐specific sample sizes for individual populations). Samples from most populations were collected across multiple years and/or consisted of multiple body size classes from a single year and so represent adults from a mixture of age classes. This is relevant to interpreting estimates of contemporary effective population size (see below). These populations were the same as those analyzed by Chiucchi and Gibbs (2010) with the difference that samples from one additional population from Spring Valley, Ohio (SPVY—see Figure 1), were analyzed using RADSeq data.

**Table 1:** Estimates of genetic variation for each population based on microsatellite and polymorphic RADSeq loci. Microsatellite results are based on data from 17 microsatellite loci reported in Chiucchi and Gibbs (2010)

Population	Code	Microsatellite					RAD					
		*N*	Ho	He	FIS	AR	*N*	*N* loci (100%)	Ho	He	Fis	AR
Southern Illinois												
South Shore State Park	SSSP	18	0.646	0.679	0.050	3.70	5	537 (59%)	0.329	0.400	0.180	1.44
Eldon Hazlet State Park	EHSP	14	0.738	0.759	0.029	4.35	8	1,183 (29%)	0.287	0.350	0.170	1.48
Western and Central Ohio												
Prairie Road Fen	PRDF	21	0.535	0.588	0.093	3.14	21	510 (43%)	0.332	0.363	0.070	1.33
Spring Valley Wildlife Area	SPVY	0	‐	‐	‐	‐	7	395 (57%)	0.340	0.395	0.127	1.32
Killdeer Plains Wildlife Area	KLDR	68	0.749	0.768	0.024	4.82	27	1,372 (25%)	0.274	0.296	0.062	1.49
Willard Marsh Wildlife Area	WLRD	15	0.676	0.663	−0.043	4.34	10	617 (60%)	0.300	0.345	0.123	1.44
Northeast Ohio												
Grand River Lowlands 1	GRL−1	18	0.576	0.571	−0.009	3.16	20	733 (32%)	0.364	0.347	−0.064	1.36
Grand River Lowlands 2	GRL−2	18	0.535	0.508	−0.055	2.79	16	533 (45%)	0.342	0.341	−0.001	1.31
Grand River Lowlands 3	GRL−3	20	0.642	0.626	−0.037	3.57	19	610 (47%)	0.321	0.330	0.020	1.37
Western Pennsylvania												
State Games Lands 95	GLAD	6	0.526	0.576	0.084	3.02	5	351 (60%)	0.373	0.402	0.074	1.27
Venango County	VNGO	7	0.532	0.606	0.132	3.55	7	410 (59%)	0.349	0.386	0.103	1.34
Jennings Environmental Education Center	JENN	9	0.595	0.688	0.143	3.58	10	410 (59%)	0.352	0.365	0.039	1.31
New York												
Bergen Swamp	BERG	20	0.558	0.545	−0.029	2.75	14	371 (50%)	0.323	0.344	0.054	1.22
Cicero Swamp	CCRO	62	0.538	0.544	0.011	2.94	27	635 (32%)	0.298	0.315	0.037	1.22
Ontario												
Bruce Peninsula Nat. Park	BPNP	20	0.708	0.720	0.016	4.46	27	886 (35%)	0.273	0.304	0.083	1.45
Beausoleil Island, Georgian Bay Is. Nat. Park	BEAU	15	0.616	0.657	0.046	3.80	22	666 (45%)	0.291	0.318	0.081	1.38
Killbear Provincial Park	KBPP	20	0.608	0.600	−0.014	3.49	18	672 (48%)	0.288	0.324	0.110	1.40

Note

Code is the identifier used for each population on Figure 1; *N* = number of individuals genotyped; Ho and He are observed and expected heterozygosity, Fis is the fixation index, and AR is allelic richness which were calculated using Arlequin (Excoffier & Lischer, 2010). N loci (100%) is the number of polymorphic RAD loci scored in individuals in that population (% scored in all individuals).

### 2.2 Genetic data

We analyzed individuals (*n* = 385) using two multilocus genetic datasets: One based on DNA microsatellite loci previously analyzed by Chiucchi and Gibbs (2010) and a new dataset based on restriction site‐associated DNA loci (RADSeq—Andrews, Good, Miller, Luikart, & Hohenlohe, 2016). Sixty percent (*n* = 229) of individuals were characterized using both genetic markers. Both datasets were used for estimating contemporary effective population sizes. Only the RADSeq dataset was used for the historical demographic analyses, which are based on modeling site frequency spectra (SFS) (see below). For the microsatellites, we used previously published data from 17 loci reported in Chiucchi and Gibbs (2010). For RADSeq loci, we generated data following the protocols described in Sovic et al. (2016). Briefly, high‐quality genomic DNA was extracted from blood or tissue samples, and double‐digest RADSeq libraries (Peterson, Weber, Kay, Fisher, & Hoekstra, 2012) were generated with EcoRI and SbfI restriction enzymes (New England Biolabs, Ipswich, MA) and a modified version of the RADSeq protocol described in DaCosta and Sorenson (2014). For details of the library preparation methods, see the Supplemental Information section of Sovic et al. (2016). Pooled libraries of up to 36 individuals were sequenced in single‐end 50‐bp runs as partial lanes on an Illumina HiSeq 2,500 sequencer. De novo locus assembly, SNP identification, and genotyping of RADSeq loci were carried out on the raw fastq data using AftrRAD version 5.0 (Sovic, Fries, & Gibbs, 2015). Unless otherwise specified, default settings were used in the AftrRAD run. Specifically, reads containing one or more bases for which the quality (Phred) score was below 20 were removed from analyses (minQual = 20). Reads that shared 90% or more sequence similarity after accounting for indels were assigned as allelic variants at a given locus as part of the de novo assembly (minIden = 90). After assembly, genotypes were called at loci for which there was a minimum of five reads in the individual (MinReads = 5). Loci with <5 reads were scored as missing data. Within each population, we then retained for analyses only those loci that were scored in all samples. Genotypes were assigned based on read (haplotype) identity for all analyses, with the exception of site frequency spectra used for model testing in fastsimcoal, which were constructed by selecting the first SNP from each locus (see details below).

### 2.3 Population structure

Repeated analyses based on DNA‐based markers such as RAPDs (Lougheed, Gibbs, Prior, & Weatherhead, 2000) and microsatellites (Chiucchi & Gibbs, 2010) have shown high levels of genetic structure and low migration rates between individual populations of *Sistrurus catenatus*. To assess whether RADSeq data showed similar levels of genetic structure, we used hierfstat (Goudet, 2005) to estimate pairwise and overall Fst for both the RADSeq dataset newly reported here and, for comparative purposes, for the microsatellite data reported by Chiucchi and Gibbs (2010). We calculated Pearson's *r* correlation and associated significance levels between pairwise Fst values generated for microsatellite and RADSeq data using the rcorr function in the R base package (R Development Core Team, 2015).

### 2.4 Effective population size

We assumed that each population was essentially genetically isolated due to high degrees of genetic structure between populations and low levels of genetically effective migration inferred by Chiucchi and Gibbs (2010). As such, we analyzed each set of samples from a single location as a genetically isolated population. We calculated contemporary effective population sizes using two different estimators for both the RADSeq and microsatellite datasets. First, we estimated contemporary Ne using the LDNe method (Waples & Do, 2008), as implemented in the program NeEstimator (Do et al., 2014). This method estimates Ne based on patterns of linkage disequilibrium between loci and was shown to perform well relative to other methods when calculating Ne under scenarios of low Ne and low migration rates (Gilbert & Whitlock, 2015). We used a “two allele” minimum for each locus within each population based on the recommendations of Waples and Do (2010) relative to the sample size of individuals (≤25) in almost all our populations. Confidence intervals for Ne values were estimated using a parametric approach implemented in the program. Second, we used Colony (Jones & Wang, 2010), which uses a maximum‐likelihood‐based method to calculate Ne based on inferred sibship frequencies among the samples, with associated confidence intervals obtained through bootstrap resampling.

### 2.5 Heterozygosity–fitness correlations

Snout–vent length (SVL) and body mass data were available for a subset (*N* = 91) of the individuals genotyped for RAD variation. These included samples from SSSP (*N* = 5), BERG (*N* = 7), CCRO (*N* = 18), GRL‐2 (*N* = 7), GRL‐3 (13), SPVY (*N* = 6), WLRD (*N* = 5), GLAD (*N* = 5), JENN (*N* = 8), VNGO (*N* = 7), and PRDF (*N* = 10). We log‐transformed (ln) these data and performed a major axis regression in the R package lmodel2 (Legende, 2018) to obtain the slope of the best‐fit line between these variables. The slope of this line (bSMA) was used to calculate the Scaled Mass Index (SMI, Peig & Green, 2009) according to the equation SMI =BMi(SVL0/SVLi)^bSMA^, where BMi is the mass of the sample, SVL0 is the mean SVL across all samples, and SVLi is the SVL of sample i. We then used major axis regression to evaluate the relationship between these SMI values and average genome‐wide SNP heterozygosity, which was estimated for each individual from RADSeq data using scripts included with AftrRAD.

### 2.6 Model‐based analyses of historical demography

**Figure 2:**
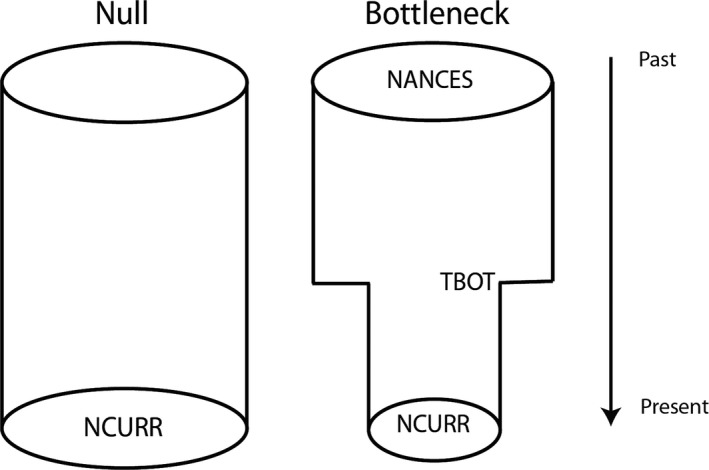
Historical demographic models analyzed for each population using fastsimcoal. Note that for the “bottleneck model,” there are no restrictions regarding the direction of the population size change, as it allows for both increases or decreases in population size. Table S3 shows the results for model comparisons for each population based on AIC. Parameter estimates for the best‐fit models are given in Table S4

We used historical demographic analyses to test between models of population history. Our goal was to assess whether evidence exists for declines in population size within each population. For declining populations, we assessed whether the declines occurred over a timescale consistent with anthropogenic impacts (arbitrarily defined as <200 years—see Discussion) or were better explained by historical factors occurring over longer timescales. To avoid problems associated with model overparameterization, we analyzed the simplest models possible that captured the key features of the process (population size change) we were interested in exploring. Specifically, we tested between two simple demographic models (Figure 2). The first (“null model”) represents a population of constant size and is defined by a single parameter, the current population size (NCURR). The second (“bottleneck model”) is characterized by three parameters: the current population size (NCURR), a time of instantaneous population size change (TBOT), and a population size prior to TBOT (NANCES). Note that although we refer to this model as the “bottleneck model,” there are no restrictions regarding the direction of the population size change, as it allows for increasing population size in addition to decreasing population size. Harris et al. (2016) used a similar approach to assess the impact of urbanization on white‐footed mice (*Peromyscus leucopus*) in New York City. Prior ranges for the three parameters “NCURR,” “NANCES,” and “TBOT” were 10–50,000, 10–50,000, and 1–10,000, respectively, and were not bounded on the upper end.

We tested among the competing historical demographic models with fastsimcoal 2.5.2 (Excoffier, Dupanloup, Huerta‐Sanchez, Sousa, & Foll, 2013), which uses maximum‐likelihood methods to perform model selection and parameter estimation based on the site frequency spectrum (sfs) calculated from the population genotypes. This approach has advantages over qualitative analyses of demography based on microsatellite data (e.g., Kimmel et al., 1998) by allowing the evaluation of the statistical fit of different demographic models using a genomic‐scale dataset summarized with a single metric. Folded site frequency spectra (calculated from observed counts of the minor allele) were generated separately for each of the 17 populations from SNPs scored in all sampled individuals. For loci with more than one variable site, only the first SNP at each locus was retained. For each of these populations, we performed 100 fastsimcoal runs (30 ECM cycles and 1e5 simulations per run) under each of the two models. The maximum‐likelihood runs under each model were then compared using AIC to select the optimal model for each population.

Once the optimal model was identified for each population, we performed additional fastsimcoal analyses to estimate parameters within each population under the optimal model. To obtain point estimates, we used site frequency spectra that included all sites, performed 100 independent fsc runs for each population, and obtained parameter estimates by selecting the values associated with the maximum‐likelihood run. To evaluate the variability of these parameter estimates, we generated 50 bootstrap resampled datasets, each with 10% fewer sites than the full dataset, from the observed sfs from each population, and performed fsc analyses on each these 50 resampled datasets (also 100 runs for each dataset; 30 ECM cycles and 1e5 simulations per run).

A key parameter in these models is the genome‐wide mutation rate. Because we are not aware of any direct estimates for genome‐wide mutation rates for snakes, we conducted a series of preliminary analyses using paired sets of models and datasets that only varied in their use of “high” and “low” mutation rates. For the “high” mutation rate, we used 2.5 × 10^−8^ per site/generation as estimated for humans (Nachman & Crowell, 2000) and used in Sovic et al. (2016). For the “low” mutation rate, we used 2.1 × 10^−9^ per site/generation as used for a recent study on frogs by Thomé and Carstens (2016) and used in Gibbs et al. (2018). We then compared the model likelihoods for each analysis and found that the use of the high mutation rate consistently generated significantly better model likelihoods (results not shown). Consequently, we used the rate of 2.5 × 10^−8^ mutations per site/generation in our subsequent analyses. Finally, as described by Sovic et al. (2016), we followed the approach of Lande, Engen, and Saether (2003) to convert estimates of time from generations to years, but used a lower estimate of age at first reproduction (2 years) which is based on data collected from the populations we studied (G. Lipps, unpublished data). This resulted in an estimated generation time of 2.03 years.

We stress that although we have conducted independent historical demographic analyses on each population the degree to which each population represents independent entity over historical timescales is unclear. Thus, as a whole the results of these analyses should be taken as a qualitative survey of the types of demographic histories experienced by an unknown number of historically independent populations across the range of this species.

### 2.7 Potential impact of Fst‐outlier loci on demographic results

Our analyses of population structure, size, and historical demography assume that the polymorphism measured represents neutral variation, yet loci showing high levels of divergence between populations (outlier loci) may represent loci under divergent selection. We explored the sensitivity of our demographic analyses to such loci in two ways. First, we used Outflank version 0.2 (Whitlock & Lotterhos, 2015) with default parameter settings to identify highly divergent loci across populations. This program represents a “second‐generation” Fst‐outlier detection program that has much lower false‐positive rates, yet comparable power as compared to earlier programs. For the Outflank dataset, we allowed up to 10% missing data at each locus. Second, because characteristics of our study system (high levels of genetic structure and small numbers of loci and populations) may make detection of outliers problematic (Lotterhos & Whitlock, 2015), we also conducted a sensitivity analyses in which we chose a set of three population pairs that represent a range of sizes and interpopulation distances, and then assessed the impact of deleting a portion of high Fst loci from the dataset on demographic estimates. Specifically, we used Genepop (Rousset, 2008) to calculate Fst for each locus in each of the following pairwise comparisons: PRDF/BERG, KLDR/KBPP, GRL‐1/GRL‐2. We then identified the loci associated with the top 5% of Fst values, removed these loci from the datasets, and used these modified datasets to calculate effective population size and perform historical demographic analyses with the same methods used for the full datasets.

### 2.8 Projecting loss of genetic variation

We assessed how existing levels of variation might change in the future for a range of contemporary effective sizes given no change in current sizes. To do this, we conducted forward genetic simulations in simuPOP with overlapping generations (Peng & Kimmel, 2005). SimuPOP is a flexible forward‐time simulator of demographic scenarios that uses life history information from the species under study. We first generated models that incorporated estimates of key features of *Sistrurus* demography (Jones et al., 2012) (see below). Next, we ran 10 iterations for each of three fixed Ne values (50, 30, and 10) that represent a range of empirically estimated values for Ne. For each iteration, 1,000 independent bi‐allelic loci were initialized for every individual with a starting frequency of *p* = 0.81 and *q* = 0.19, corresponding to a starting He of 0.30, which approximates the currently observed He in polymorphic RADSeq loci (Table 1). Simulations were run for 100 time steps, where one time step equaled one year. Individuals did not reproduce until year 2 and died after year 5. We based our choices of minimum reproductive ages and maximum ages based on previously published demographic data. While *S. catenatus* may live up to 12 years, less than 70% of a breeding cohort is expected to reach age 5 (mean annual survival of 0.67; Jones et al., 2012). During each time step, individuals were aged by one year, and those greater than 5 years old removed from the simulated population. Random pairs of adults in the population were then chosen to reproduce until the population returned to the fixed population size. After new individuals were generated, individual heterozygosity was exported every time step for later analysis. For each run, individual heterozygosity was used to calculate the average heterozygosity for each year. Standard deviation of the observed heterozygosity was calculated for each year from the 10 iterations and used to generate 95% confidence intervals.

We compared our simulation results with a widely used analytical approach for estimating loss of variation based on population genetic theory (James, 1970) that has been used for estimating changes in heterozygosity in endangered species (Amos & Balmford, 2001). Specifically, James (1970) proposed using the equation Ht = H0 (1−1/2Ne)*^t^* to describe changes in heterozygosity over time in bottlenecked populations where H0 is initial heterozygosity and Ht is heterozygosity t generations after the instantaneous decline of a large population to size Ne. A key difference between the two methods is that compared to the simulation‐based approach in SimuPOP, this analytical approach assumes no overlapping generations with all individuals only reproducing in a single generation. As with our simuPOP runs, we fixed the initial H0 at 0.3 and then projected the loss of variation using the equation above for three fixed values of Ne: 50, 30, and 10.

## 3 RESULTS

### 3.1 Genetic markers

For the RADSeq data, a mean of 1,037,766 sequence reads were assigned to each of the 263 samples (range 216,381–4,239,460). The average numbers of total non‐paralogous loci and polymorphic loci per population were 52,815 and 641, respectively (Table 1). For a given population, the median read depth ranged from 48 to 61 reads per polymorphic locus, and the percentage of polymorphic loci that were scored for all sampled individuals ranged from 25% to 60% across the 17 datasets (Table 1). These values for coverage are consistent with those reported in Sovic et al. (2016), while the number of polymorphic loci used in the analyses here is smaller than for the previous study. This is because datasets in this study represent only intra‐population variation, while the datasets for the previous study included both intra‐ and interpopulation variation.

### 3.2 Genetic structure

The overall Fst for RADSeq data across all populations was 0.283 with pairwise values for individual populations ranging from 0.083 to 0.398 (Table S1a). These values are similar to values estimated from microsatellite data (overall value: 0.223; range of pairwise values: 0.088–0.51; Table S1b). Fst values based on RADSeq and microsatellite data for specific pairwise comparisons are significantly correlated with each other (*r* = 0.74; *p* < 0.05) with microsatellite‐based values averaging ~22% less than matched RADSeq‐based values. The variation in pairwise values is similar, with coefficients of variation of 28.1% and 31.6% for pairwise Fst values based on RADSeq and microsatellite data, respectively. We note that direct comparisons of Fst values based on multi‐allelic microsatellite loci and bi‐allelic SNP loci are problematic because differences in Fst based on each type of marker do not scale in the same way (Frankham et al., 2017). However, qualitatively, the RADSeq data support previous results from the microsatellite analyses of Chiucchi and Gibbs (2010) that these populations are genetically distinct and demographically isolated from each other.

### 3.3 Estimates of effective population size

**Figure 3:**
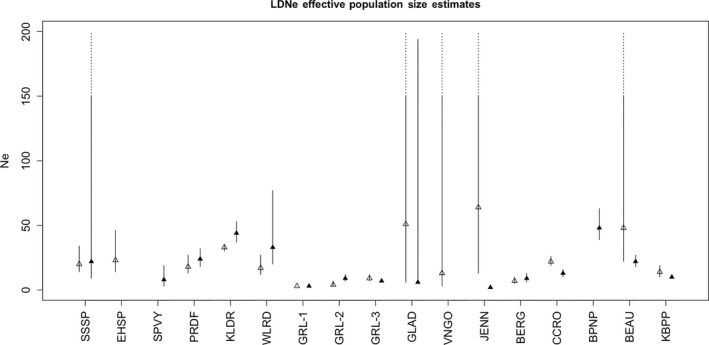
Contemporary effective population size estimates based on LDNe generated from microsatellites (hollow triangles) and RADSeq data (filled triangles) for 17 populations of s. catenatus. Confidence intervals with an upper bound of infinity are represented by dashed lines extending to the top of the plot. Only RADSeq data are available for the population from SPVY

Point estimates of the effective population sizes for individual populations vary depending on the type of genetic data and method used to generate the estimates (Figure 3; Table S2). A small number of populations (RADSeq: EHSP and VNGO; microsatellites: BPNP and GLAD) yielded estimates that were 1–2 orders of magnitude greater than other populations or were estimated as infinite in size—these are coded as NA in Table S2, and omitted from Figure 3. Estimates from LDNe for RADSeq data range between 2 (JENN) to 48 (BPNP) with an overall mean (±*SE*) of 17.3 ± 3.5. Microsatellite‐based estimates of LDNe ranged from 3 (GRL‐1) to 64 (JENN) with an overall mean that is slightly higher (20.6 ± 3.6 *SE*). For estimates based on RADSeq data, all populations had values <50, whereas for microsatellite data, 13 of 15 (87%) populations were below this threshold. These summaries include all populations including those where estimates were not available for one type of data. When paired estimates for single populations are compared, mean values based on RADSeq data are slightly lower (RADSeq: 15.6 ± 3.5; microsatellites: 21.1 ± 5). Paired values based on each type of data are not significantly correlated with each other (*r* = 0.11; *p* > 0.05).

For sibship frequency‐based measures of Ne, point estimates range from 8 (SPVY) to 140 (BPNP) (mean: 36.38 ± 10.2) for RADSeq data, and 14 (GRL‐1) to 95 (BPNP) (mean: 44.06 + 5.8) for microsatellite data. Proportionally fewer populations (RADSeq: 77% [10/13]; microsatellites: 75% [12/16]) had sibship‐based values <50. Paired estimates show similar patterns (RADSeq: mean = 29.55 ± 8.5; microsatellites: 35.36 ± 10.2) but, in contrast to LDNe estimates, are highly correlated with each other (*r* = 0.89; *p* < 0.05). In general, although estimates of contemporary Ne vary depending on method and marker type, most populations have small effective sizes of <50 with only a few populations having larger sizes.

### 3.4 Heterozygosity–fitness correlations

There was no significant relationship between our estimate of individual fitness (standardized mass index−SMI) and genome‐wide individual heterozygosity estimates based on RADSeq data (*r* ^2^ = 0.001; *p* = 0.418). We also performed regression analyses on individual populations with a minimum of five samples, but as for the full dataset, no significant positive relationships were detected (results not shown).

### 3.5 Demographic modeling

Statistical comparisons of the two demographic models in Figure 2 show strong support (AIC ~1) for the model incorporating population size change as best‐fitting the data for all individual populations (Table S3). Estimates of ancestral population size (Na) averaged 39,197 (range 21,073–56,684) and were consistently higher than those for current population size (Nc), which had a mean of 2,445 (range 10–12,583), suggesting all populations were best modeled as having experienced a population decline.

**Figure 4:**
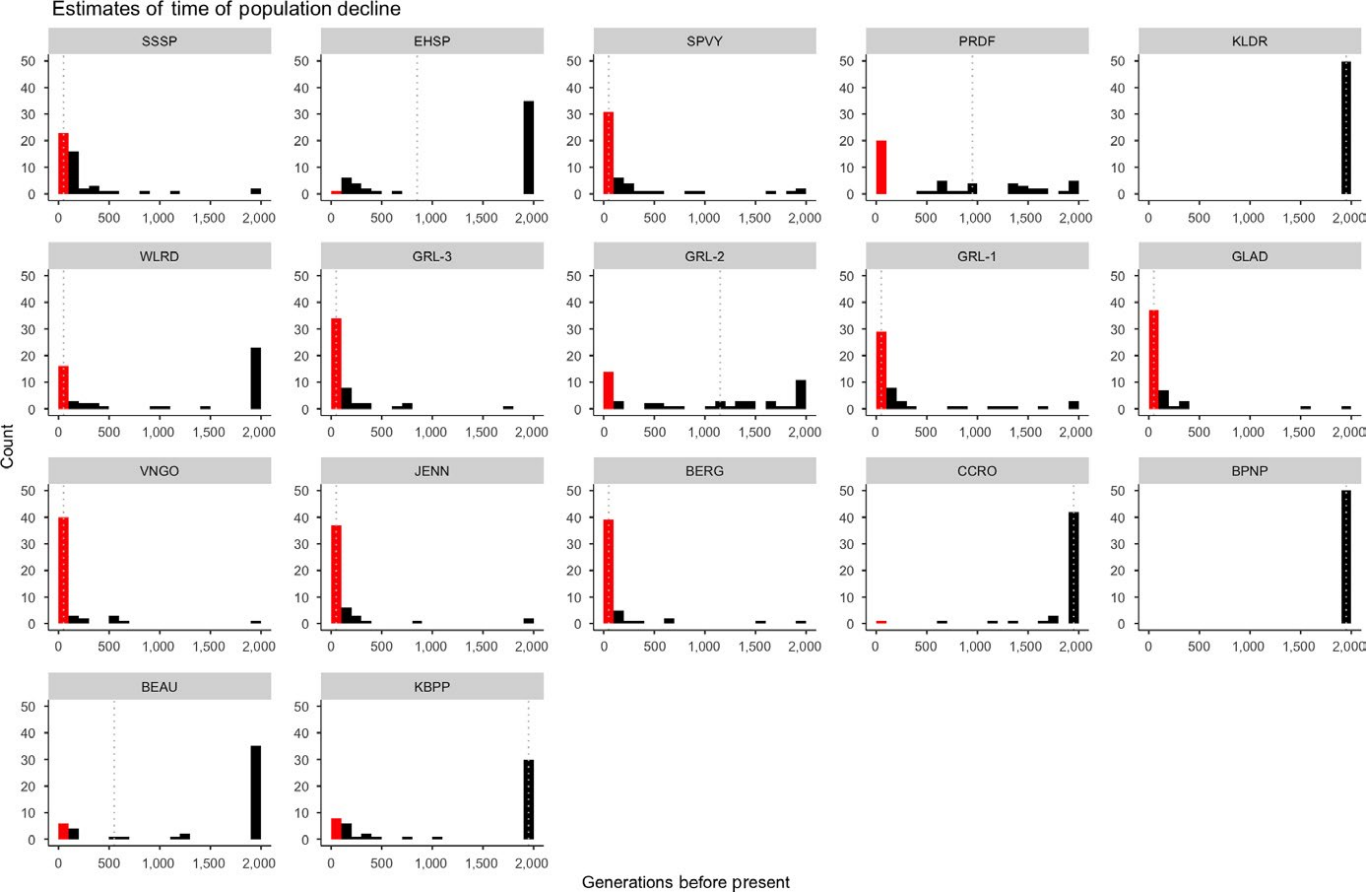
Histograms representing maximum‐likelihood estimates from fastsimcoal of the number of generations in the past at which a decline in population size occurred for each of the 17 populations. The dotted gray line indicates the bin containing the point estimate (see Table S4), and distributions reflect estimates from 50 bootstrapped datasets for each population. Times along the x‐axis are binned into intervals of 100 generations. The bin of 0–100 generations, roughly corresponding to an anthropogenic timeframe (~200 years), is indicated with red. All estimates exceeding 2,000 generations are combined into the 2,000 generation bin

In terms of the timing of this decline, joint inspection of the point estimates relative to the parameter distributions from the resampled datasets shows two scenarios (Figure 4). For 12 populations, there is a close correspondence between the point estimate of TBOT (in generations) from the original data and the distribution of TBOT values from the resampled data. In contrast, for five populations (EHSP, PRDF, WLRD, GRL‐2, and BEAU), the point estimate does not mirror the resampled distribution, and therefore, TBOT cannot be inferred with confidence. For populations with coincident point estimates and distributions, eight populations have small values (<100 generations) suggesting recent declines within the past 200 years (SSSP, SPVY, GRL‐1, GRL‐3, GLAD, VNGO, JENN, and BERG), while four populations have relatively large values (>2,000 generations or>4,000 years) suggesting declines on historical timescales. Thus, for populations for which TBOT can be inferred with confidence, a majority (8/12; 67%) showed declines during the time period in which humans have had strong impacts on the landscape, while four showed declines on historical timescales.

### 3.6 Lack of impact of Fst‐outlier loci on demographic estimates

None of the 701 loci included in the Outflank analysis of the 17 populations were flagged as Fst‐outliers. Sensitivity analyses for six populations suggested that removal of loci associated with the top 5% of Fst values based on pairwise population comparisons had little effect on our results. Specifically, for all six populations, estimates of LDNe, and model choice results and parameter estimates from fsc were identical or very similar to those from the full dataset (results not shown). These findings indicate that even if our datasets contain subsets of loci under divergent selection not detected by our Fst‐outlier tests due to a lack of power, it is unlikely such loci are having an impact on our demographic inferences.

### 3.7 Projected loss of variation

**Figure 5:**
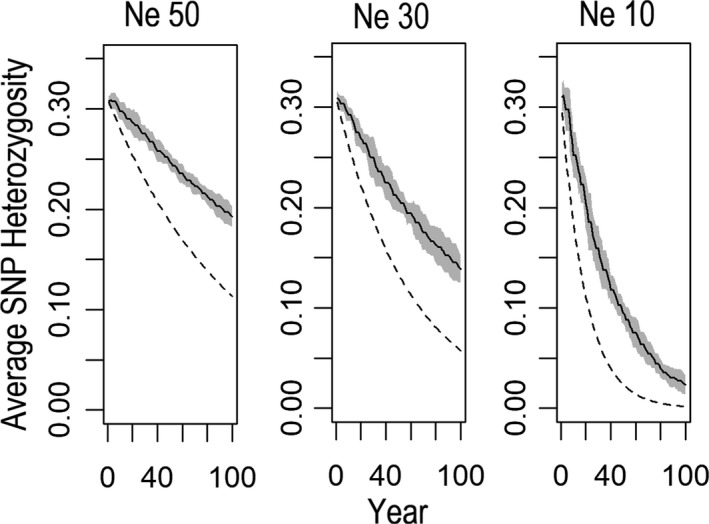
Results of simulations showing the projected loss of existing levels of variation (estimated as He) over time for populations of varying effective size. Trajectories are shown for fixed Ne values of 50, 30, and 10 individuals from results based on the SimuPOP simulations (point estimates shown by solid line +95% CI polygons) or the analytical formula for loss of He over time in a bottlenecked population

All simulated populations lost variation over time, with the greatest loss occurring in the simulation based on the smallest fixed value of Ne (Figure 5). Based on point estimates of He at 0, 30, and 50 generations, populations with an Ne value of 50 lost 21% of their initial variation, those with Ne = 30 lost 33% of their variation, and populations with Ne = 10 lost 68% of their variation over roughly 100 years given our estimate of the generation time of this snake. These estimates of expected loss of He were substantially less than the predicted losses over the same period of time based on the analytical formula that assumes no overlapping generations, which showed declines of He that varied from 63% (Ne = 50) to 99% (Ne = 10) (Figure 5). This demonstrates the importance of incorporating realistic demographic parameters into models used for projecting loss of variation. Overall, these results suggest that nearly all populations are not at genetic equilibrium relative to their current effective size but have excess polymorphism which they will lose due to drift at varying rates over the next 100 years. This implies that most have undergone recent declines in census size (reflected in estimates of current effective size), but these declines have yet to influence the standing genetic variation present in a given population.

## 4 DISCUSSION

### 4.1 Estimation of contemporary Ne

A major result is that point estimates of contemporary Ne are small (<50) for the majority of populations sampled from across the range of *Sistrurus catenatus*. For example, estimates based on LDNe analysis of RADSeq data show that mean Ne is ~22 individuals (range: 2–48), although the confidence estimates for individual populations can be large. This result of small Ne values holds regardless of the method used to generate the estimates (i.e., LDNe vs. Colony), or the type of genetic marker (microsatellites vs. RADSeq), and suggests that genetic drift is poised to have a significant impact on the maintenance of genetic variation in these populations.

These estimates are much smaller than the effective sizes based on microsatellite variation reported by Gibbs and Chiucchi (2012) for many of the same populations reported here and, in some cases, for estimates of Ne based on historical demographic modeling of RADSeq‐based polymorphism by Sovic et al. (2016) (and here). This is because these studies estimate Ne over different timescales using different methodologies, and so values from each set of studies are not directly comparable (Hare et al., 2011). For example, the estimates of Ne in Chiucchi and Gibbs (2010) are based on results from Migrate (Beerli, 2009) which generates estimates of long‐term effective size. These reflect the amount of genetic diversity (estimated as)maintained in a population at evolutionary equilibrium due to the joint effect of drift and mutation (µ) over a period of approximately 4Ne generations ( = 4Neµ). Likewise, the estimates of Ne using *fastsimcoal* generated by Sovic et al. (2016) and here also represent historical estimates of effective population size over many past generations. These long‐term estimates have conservation value in potentially representing a measure of population size that predates human impacts. As such, they could represent a historical target for assessing current management goals (Hare et al., 2011) or for evaluating the magnitude of recent anthropogenic impacts relative to historical population sizes (Alter, Rynes, & Palumbi, 2007). Long‐term estimates can also be useful for species‐level comparisons relevant to conservation. For example, Sovic et al. (2016) compared long‐term effective sizes and population dynamics of *S. catenatus* with the closely related but non‐threatened Western Massasauga Rattlesnake (*S. tergeminus*). The demographic patterns they detected were consistent with differences in the conservation status of each species. The long‐term effective population size was much smaller for the representative *S. catenatus* population than for the *S. tergeminus* population, and the *S. tergeminus* population was best modeled as a growing population, whereas *S. catenatus* showed evidence of a long‐term population decline.

In contrast, the short‐term estimates of Ne based on single sample estimators reported here measure effective population size over much more recent timescales—approximately the period of sample collection, and hence are useful for different conservation purposes (Hare et al., 2011). Specifically, in species with overlapping generations, they generate an estimate of Ne that is approximately equal to Nb * generation time, where Nb is the effective number of breeding individuals in a given year. As such, short‐term estimates are more useful for estimating short‐term abundance using empirically derived estimates of the ratio of Ne/Nc, where Nc is population census size (Palstra & Ruzzante, 2008), and evaluating the degree to which genetic drift may erode the adaptive genetic potential of small populations over timescales relevant to management activities (Lynch, Conery, & Burger, 1995).

Because our analyses use mixtures of age classes of individuals from each population, they may underestimate true values of contemporary Ne (Waples, Antao, & Luikart, 2014). The degree of this bias is related to the ratio of adult lifespan/generation time, which we estimate as ~4. This assumes a two‐year generation time (see above) and uses the range‐wide estimate of annual survivorship (0.67—Jones et al., 2012) to project when 95% of a hypothetical cohort of snakes would have died. Given this value, extrapolations from the trends shown in Figure 6 in Waples et al. (2014) suggest that our estimates of Ne based on LDNe may be underestimated by as much as 25%.

Two other studies have recently reported contemporary effective size estimates for this species using microsatellite genetic data analyzed using the LDNe program. Both show estimates broadly similar to the values reported in this paper. Bradke et al. (2018) reported values of 29.5 (95% CI = 22.2–40.5) and 44.2 (95% CI = 29.7–73.4) for two populations in Michigan. Baker et al. (2018) reported Ne values of ~30 individuals (95% CIs ~15–47) for each of three point estimates over a 10‐year period (2002 to 2012) for the SSSP population in Illinois reported here. As described by Bradke et al. (2018), these values fall within the range of LDNe estimates found for other threatened and non‐threatened snakes (see Table 3 in Bradke et al. (2018)).

A general benchmark for evaluating whether species are at short‐term genetic risk comes from the widely debated “50/500 rule” first proposed by Franklin (1980) for assessing the minimum viable size for endangered species. Briefly, the rule proposed that to avoid the deleterious effects of inbreeding depression, populations should be maintained at a minimum short‐term effective size of at least 50 individuals. The validity of 50 individuals as a specific numerical target has been much discussed (Frankham, Bradshaw, & Brook, 2014; Jamieson & Allendorf, 2012) and argued to be too small to achieve its purpose (Frankham et al., 2014). Regardless, our empirical finding that most populations of *S. catenatus *have a contemporary Ne of <50 suggests that these populations are potentially at risk of the deleterious effects of inbreeding depression (but see Wood, Yates, & Fraser, 2016) and that conservation planning for this species needs to take this possibility into account.

### 4.2 Heterozygosity–fitness correlations

Our results reinforce those of Gibbs and Chiucchi (2012) who also found little evidence of genetic costs of inbreeding based on heterozygosity–fitness correlations (HFCs) (Chapman et al., 2009). We note that the datasets used in the two studies are not independent. Many of the same individuals (and their estimates of body condition) were used in both analyses, with the difference being in the type and number of genetic loci used to estimate individual heterozygosity. We echo the cautions of Gibbs and Chiucchi (2012) that body condition is an indirect measure of individual fitness in these animals and that small sample sizes combined with small effect sizes may reduce the statistical power of both analyses to detect effects. A more direct way to test whether populations are currently experiencing significant mutational load would be future genome‐level assessments of whether deleterious mutations such as loss of functional variants (e.g., Rogers & Slatkin, 2017) occur in individuals, and whether the frequency of such variants is greater in small versus large populations (Perrier, Ferchaud, Sirois, Thibault, & Bernatchez, 2017).

### 4.3 Historical demography

Our analysis of historical demography suggests that range‐wide, all populations have undergone a decline in population size. However, based on point estimates, the timing of the population reduction varies: Eight populations show a decline within the past 200 years, whereas four others experienced declines >2,000 years bp. This suggests that there are two different classes of drivers for the population declines. The first operates on contemporary timescales that are coincident with the colonization and subsequent landscape modification by European settlers in North America, which has occurred within the past 200 years (Pielou, 1991; Schmidt, 1938). In contrast, the older dates correspond with large‐scale environmental changes related to climate that have previously been hypothesized to impact the distribution of this (Cook, 1992) and other species in this region of North America (Soltis, Morris, McLachlan, Manos, & Soltis, 2006).

There are no obvious spatial patterns in populations showing recent versus historical declines that might suggest a broad‐scale geographic cause to such variation. Populations within the same geographic regions show either different (e.g., EHSP and SSSP) or similar (e.g., VNGO, GLAD, and JENN) timescales for declines. This lack of a pattern suggests that local conditions may play a primary role in determining the timescale of population declines. This hypothesis could be explored by incorporating a historical dimension to evaluating habitat suitability in the local area where each population is found (e.g., McClusky et al., 2018).

These results are at odds with previous work that showed limited evidence for population declines in these populations using different methods of analyses and microsatellite genetic data. Specifically, Chiucchi and Gibbs (2010) used allele distribution tests implemented in Bottleneck (Luikart, Allendorf, Cornuet, & Sherwin, 1998) and only found significant results supporting recent bottlenecks in three of 16 populations. We suggest that the SFS‐based tests used here are more sensitive because they employ an explicit model‐testing framework, use a much greater amount of genetic data, and examine the possibility of declines over a broader timescale.

Other studies using similar approaches have found evidence for either recent or historical bottlenecks in other endangered vertebrates (Dussex, Rawlence, & Robertson, 2015; Goossens et al., 2006; Salmona et al., 2012; Tucker et al., 2012; Zhu et al., 2013), but in general, the focus is on one or a few populations of each species. Our study differs in that it examines evidence for declines across a relatively large number of independent populations, and shows that the timing of significant declines varies across populations. This suggests that characterizing species as impacted only at historical or contemporary timescales is simplistic and that a given species may experience both types of declines. Identifying populations that have experienced significant recent declines may represent a way of prioritizing certain populations over others in terms of the impacts of anthropogenic effects on the viability of specific populations.

### 4.4 Projecting future levels of genetic variability

Our simulations suggest that if there are no changes in key population parameters like size and levels of migration, most *Sistrurus catenatus* populations will lose 20% or more of their existing variation over the next 100 years. These conclusions are based on several assumptions. First, they assume populations will remain no larger than their currently estimated effective size with no increase in migration. This is reasonable given that trends over time are for populations to remain isolated with the same or declining numbers Szymanski et al.., 2016). Second, our analyses are based on the evolutionary dynamics of what we assume is neutral variation and not the adaptive variation that is used to delineate adaptive conservation units within threatened taxa (Funk, McKay, Hohenlohe, & Allendorf, 2012). This is a common problem in conservation genetic studies in that the genes that underlie adaptive variation are difficult to identify. However, Reed and Frankham (2003) have provided evidence that the two types of variation are broadly correlated with each other. In *Sistrurus*, studies of adaptive variation within populations are scarce, but Jaeger et al. (2016) have documented variation in MHC loci in three populations in Illinois that showed qualitative association with variation in six microsatellite loci. In addition, Ochoa et al. (in prep.) have found evidence for polymorphism in genes associated with venom proteins. In general, these findings suggest that adaptive variation is present in existing populations of *Sisturus *and that high levels of drift have yet to purge this variation. However, whether the rate at which it is expected to be lost parallels the results of our simulations is unclear. Because selection generally acts to retard the impact of drift on the loss of variation within populations, we suspect that the timeline for loss of variation established by our simulations may be conservative.

These results have conservation implications. They suggest that existing populations of *Sistrurus* may not yet have paid the true genetic cost of living in their current size population, but that this cost could be “paid” in the near future. As such, despite their history of living in small populations over historical timescales (Chiucchi & Gibbs, 2010), they may be moving to a new equilibrium with respect to gene dynamics, with issues to do with losses of adaptive genetic variation becoming more prominent in the near future. In other words, many of these populations may be poised to enter the extinction vortex (Gilpin & Soulé, 1986) but have yet to experience the fitness costs that will act in a positive feedback loop to drive populations to even smaller sizes. The simulations may also provide a timeline that could guide the timing of introductions associated with genetic rescue (Tallmon et al., 2004) in the event it is adopted as a viable management option (Ralls et al., 2017). Specifically, our simulations suggest that as a guide to preserve 50% of the existing variation in the very smallest populations (Ne = 10), new genetic variation would need to be introduced within the next 40 years, whereas for the largest populations (Ne = 50), this could be delayed for as long as >100 years. Genetic introductions have been used to increase population viability in other highly inbred snake populations through the introduction and subsequent removal of radio‐tagged males (Madsen, Shine, Olsson, & Wittzell, 1999; Madsen, Ujvari, & Olsson, 2004). We feel that a similar approach could be used to supplement variation in populations of *Sistruru*s, especially given the fact that this species represents a taxon where outbreeding depression is not expected to be a significant issue (Frankham, 2015).

## CONFLICT OF INTEREST

None declared.

## Data Availability

Raw fastq data files used in the genetic analyses are available from the Dryad Digital Repository: https://doi.org/10.5061/dryad.jg81r80.

## References

[eva12731-bib-0001] Allendorf, F. W. , Luikart, G. H. , & Aitken, S. N. (2013). Conservation and the genetics of populations, 2nd ed. Hoboken, NJ: Wiley.

[eva12731-bib-0002] Alter, S. E. , Rynes, E. , & Palumbi, S. R. (2007). DNA evidence for historic population size and past ecosystem impacts of gray whales. Proceedings of the National Academy of Sciences, 104, 15162–15167. 10.1073/pnas.0706056104 PMC1975855 17848511

[eva12731-bib-0003] Amos, W. , & Balmford, A. (2001). When does conservation genetics matter? Heredity, 87, 257–265. 10.1046/j.1365-2540.2001.00940.x 11737272

[eva12731-bib-0004] Andrews, K. R. , Good, J. M. , Miller, M. R. , Luikart, G. , & Hohenlohe, P. A. . (2016). Harnessing the power of RADseq for ecological and evolutionary genomics. Nature Reviews Genetics, 17, 81–92. 10.1038/nrg.2015.28 PMC4823021 26729255

[eva12731-bib-0005] Baker, S. J. , Anthonysamy, W. J. B. , Davis, M. A. , Dreslik, M. J. , Douglas, M. R. , Douglas, M. E. , & Phillips, C. A. (2018). Temporal patterns of genetic diversity in an imperiled population of the Eastern Massasauga Rattlesnake (*Sistrurus catenatus*). Copeia, 106, 414–420.

[eva12731-bib-0006] Beerli, P. (2009). How to use MIGRATE or why are Markov chain Monte Carlo programs difficult to use In Bertorelli G., Bruford M. W., Hauffe H. C., Rizzoli A., & Vernesi C. (Eds.), Population genetics for animal conservation (pp. 42–79). Cambridge, UK: Cambridge University Press.

[eva12731-bib-0007] Bradke, D. R. , Hileman, E. T. , Bartman, J. F. , Faust, L. J. , King, R. B. , Kudla, N. , & Moore, J. A. (2018). Implications of small population size in a threatened pitviper species. Journal of Herpetology. inpress

[eva12731-bib-0008] Busch, J. D. , Waser, P. M. , & DeWoody, J. A. (2007). Recent demographic bottlenecks are not accompanied by a genetic signature in two populations of banner‐tailed kangaroo rats (*Dipodomys spectabilis*). Molecular Ecology, 16, 2450–2462. 17561905 10.1111/j.1365-294X.2007.03283.x

[eva12731-bib-0009] Carvajal‐Rodríguez, A. (2010). Simulation of genes and genomes forward in time. Current Genomics, 11, 58–61. 20808525 10.2174/138920210790218007 PMC2851118

[eva12731-bib-0010] Chapman, J. R. , Nakagawa, S. , Coltman, D. W. , Slate, J. , & Sheldon, B. C. (2009). A quantitative review of heterozygosity‐fitness correlations in animal populations. Molecular Ecology, 18, 2746–2765. 10.1111/j.1365-294X.2009.04247.x 19500255

[eva12731-bib-0011] Charlesworth, D. , & Willis, J. H. (2009). Fundamental concepts in genetics: The genetics of inbreeding depression. Nature Reviews Genetics, 10, 783–796. 10.1038/nrg2664 19834483

[eva12731-bib-0012] Chiucchi, J. E. , & Gibbs, H. L. (2010). Similarity of contemporary and historical gene flow among highly fragmented populations of an endangered rattlesnake. Molecular Ecology, 19, 5345–5358. 10.1111/j.1365-294X.2010.04860.x 20964755

[eva12731-bib-0013] Cook, F. R. (1992). After an ice age: Zoogeography of the massasauga within a Canadian herpetofaunal perspective In Johnson B., & Menzies V. (Eds.), International Symposium and Workshop on the Conservation of the Eastern Massasauga Rattlesnake (pp. 19–25). Toronto, ON: Zoo.

[eva12731-bib-0014] DaCosta, J. M. , & Sorenson, M. D. (2014). Amplification biases and consistent recovery of loci in a double‐digest RAD‐seq protocol. PLoS ONE, 9, e106713 10.1371/journal.pone.0106713 25188270 PMC4154734

[eva12731-bib-0015] Development Core Team (2015). R: A language and environment for statistical computing. Vienna, Austria: R Foundation for Statistical Computing.

[eva12731-bib-0016] Do, C. , Waples, R. S. , & R.S., Peel, D., Macbeth, G.M., Tillett, B.J., and Ovenden, J.R., (2014). NeEstimator v2: Re‐implementation of software for the estimation of contemporary effective population size (Ne) from genetic data. Molecular Ecology Resources, 14, 209–214. 23992227 10.1111/1755-0998.12157

[eva12731-bib-0017] Dussex, N. , Rawlence, N. J. , & Robertson, B. C. (2015). Ancient and contemporary DNA reveal a pre‐human decline but no population bottleneck associated with recent human persecution in the Kea (*Nestor notabilis*). PLoS ONE, 10, e0118522 10.1371/journal.pone.0118522 25719752 PMC4342260

[eva12731-bib-0018] Excoffier, L. , & Lischer, H. E. L. . (2010) Arlequin suite ver 3.5: A new series of programs to perform population genetics analyses under Linux and Windows. Molecular Ecology Resources, 10, 564–567. 21565059 10.1111/j.1755-0998.2010.02847.x

[eva12731-bib-0019] Excoffier, L. , Dupanloup, I. , Huerta‐Sanchez, E. , Sousa, V. C. , & Foll, M. (2013). Robust demographic inference from genomic and SNP data. PLOS Genetics, 9, e1003905 10.1371/journal.pgen.1003905 24204310 PMC3812088

[eva12731-bib-0020] Frankham, R. (2015). Genetic rescue of small inbred populations: Meta‐analysis reveals large and consistent benefits of gene flow. Molecular Ecology, 24, 2610–2618. 10.1111/mec.13139 25740414

[eva12731-bib-0021] Frankham, R. , Ballou, J. D. , Ralls, K. , Eldridge, M. D. B. , Sunnucks, P. , Frankham, R. , & Lacy, R. C. (2017). Genetic management of fragmented animal and plant populations. Oxford, UK: Oxford University Press.

[eva12731-bib-0022] Frankham, R. , Bradshaw, C. J. A. B. , & Brook, B. W. (2014). Genetics in conservation management: Revised recommendations for the 50/500 rules, Red List criteria and population viability analyses. Biological Conservation, 170, 56–63. 10.1016/j.biocon.2013.12.036

[eva12731-bib-0023] Franklin, I. R. (1980). Evolutionary change in small populations In Soulé M. E., Wilcox B. A., (Eds.), Conservation biology: an evolutionary‐ecological perspective. (pp. 135–150). Sunderland, Massachusetts: Sinauer

[eva12731-bib-0024] Funk, W. C. , McKay, J. K. , Hohenlohe, P. A. , & Allendorf, F. W. (2012). Harnessing genomics for delineating conservation units. Trends in Ecology and Evolution, 27, 489–496. 10.1016/j.tree.2012.05.012 22727017 PMC4185076

[eva12731-bib-0025] Garza, J. C. , & Williamson, E. G. (2001). Detection of reduction in population size using data from microsatellite loci. Molecular Ecology, 10, 305–318. 11298947 10.1046/j.1365-294x.2001.01190.x

[eva12731-bib-0026] Gibbs, H. L. , & Chiucchi, J. E. (2012). Inbreeding, body condition, and heterozygosity‐fitness correlations in isolated populations of the endangered eastern massasauga rattlesnake (*Sistrurus c. catenatus*). Conservation Genetics, 13, 1133–1143. 10.1007/s10592-012-0360-z

[eva12731-bib-0027] Gibbs, H. L. , Sovic, M. , Amazonas, D. , Salazar-Valenzuela, D. , Chalkidis, H. , & Moura-da-Silva, A. (2018). Recent lineage diversification in a venomous snake through dispersal across the Amazon River. Biological Journal of the Linnean Society, 123, 651–665.

[eva12731-bib-0028] Gilbert, K. J. , & Whitlock, M. C. (2015). Evaluating methods for estimating local effective population size with and without migration. Evolution, 69, 2154–2166. 10.1111/evo.12713 26118738

[eva12731-bib-0029] Gilpin , M. E. , & Soulé , M. E. (1986). In Soulé M. E. (Ed.), (pp. 19–34). Sunderland, MA: Sinauer.

[eva12731-bib-0030] Goossens, B. , Chikhi, L. , Ancrenaz, M. , Lackman‐Ancrenaz, I. , Andau, P. , & Bruford, M. W. (2006). Genetic signature of anthropogenic population collapse in orang‐utans. PLoS Biology, 4, e25 10.1371/journal.pbio.0040025 16417405 PMC1334199

[eva12731-bib-0031] Goudet, J. (2005). hierfstat, a package for r to compute and test hierarchical F‐statistics. Molecular Ecology Resources, 5, 184–186.

[eva12731-bib-0032] Government of Canada (2009). Species at Risk Public Registry. https://www.sararegistry.gc.ca.

[eva12731-bib-0033] Hare, M. P. , Nunney, L. , Schwartz, M. , Ruzzante, D. , Burford, M. , Waples, R. , … Palstra, F. (2011). Understanding and estimating effective population size for practical applications in marine species management. Conservation Biology, 25, 438–449. 21284731 10.1111/j.1523-1739.2010.01637.x

[eva12731-bib-0034] Harris, S. E. , Xue, A. T. , Alvarado Serrano, D. , Boehm, J. T. , Joseph, T. , Hickerson, M. J. , & Munshi‐South, J. (2016). Urbanization shapes the demographic history of a native rodent (the white‐footed mouse, Peromyscus leucopus) in New York City. Biology Letters, 12, 20150983. 27072402 10.1098/rsbl.2015.0983 PMC4881337

[eva12731-bib-0035] Hoffman, J. I. , Grant, S. M. , Forcada, J. , & Phillips, C. D. (2011). Bayesian inference of a historical bottleneck in a heavily exploited marine mammal. Molecular Ecology, 20, 3989–4008. 10.1111/j.1365-294X.2011.05248.x 21895820

[eva12731-bib-0036] Husemann, M. , Zachos, F. E. , Paxton, R. J. , & Habel, J. C. (2016). Effective population size in ecology and evolution. Heredity, 117, 191–192. 10.1038/hdy.2016.75 27553454 PMC5026761

[eva12731-bib-0037] Jaeger, C. P. , Duvall, M. R. , Swanson, B. J. , Phillips, C. A. , Dreslik, M. J. , Baker, S. J. , & King, R. B. (2016). Microsatellite and major histocompatibility complex variation in an endangered rattlesnake, the Eastern Massasauga (*Sistrurus catenatus*). Ecology and Evolution, 6, 3991–4003. 27516858 10.1002/ece3.2159 PMC4874855

[eva12731-bib-0038] James, J. W. (1970). The founder effect and response to artificial selection. Genetic Resources, 16, 241–250. 10.1017/S0016672300002500 5512250

[eva12731-bib-0039] Jamieson, I. G. , & Allendorf, F. (2012). How does the 50/500 rule apply to MVPs? Trends Ecology Evolution, 27, 578–584. 10.1016/j.tree.2012.07.001 22868005

[eva12731-bib-0040] Jones, O. R. , & Wang, J. (2010). COLONY: A program for parentage and sibship inference from multilocus genotype data. Molecular Ecology Resources, 10, 551–555. 10.1111/j.1755-0998.2009.02787.x 21565056

[eva12731-bib-0041] Jones, P. C. , King, R. B. , Bailey, R. L. , Bieser, N. D. , Bissell, K. , Campa, H. … Yagi, A. (2012). Range‐wide analysis of eastern massasauga survivorship. The Journal of Wildlife Management, 76, 1576–1586. 10.1002/jwmg.418

[eva12731-bib-0042] Kimmel, M. , Chakraborty, R. , King, J. P. , Bamshad, M. , Watkins, W. S. , & Jorde, L. B. (1998). Signatures of population expansion in microsatellite repeat data. Genetics, 148, 1921–1930. 9560405 10.1093/genetics/148.4.1921 PMC1460085

[eva12731-bib-0043] Lande, R. , Engen, S. , & Saether, B. E. . (2003). Stochastic Population Dynamics in Ecology and Conservation. Oxford, UK: Oxford University Press.

[eva12731-bib-0044] Lawton‐Rauh , A. (2008). Demographic processes shaping genetic variation. Current Opinion in Plant Biology, 11, 103–109. 10.1016/j.pbi.2008.02.009. 18353707

[eva12731-bib-0045] Legende, P. (2018). *Lmodel2: Model II regression* . R package version 1.7‐3.

[eva12731-bib-0046] Lotterhos, K. E. , & Whitlock, M. C. (2015). The relative power of genome scans to detect local adaptation depends on sampling design and statistical method. Molecular Ecology, 24, 1031–1046. 25648189 10.1111/mec.13100

[eva12731-bib-0047] Lougheed, S. C. , Gibbs, H. L. , Prior, K. A. , & Weatherhead, P. J. (2000). The relative utility of RAPD versus microsatellite DNA markers for assessing population structure in the Eastern Massasauga Rattlesnake. Journal of Heredity, 91, 458–463. 11218083 10.1093/jhered/91.6.458

[eva12731-bib-0048] Luikart, G. , Allendorf, F. W. , Cornuet, J. M. , & Sherwin, W. B. (1998). Distortion of allele frequency distributions provides a test for recent population bottlenecks. Journal of Heredity, 89, 238–247. 10.1093/jhered/89.3.238 9656466

[eva12731-bib-0049] Luikart, G. , Ryman, N. , Tallmon, D. A. , Schwartz, M. K. , & Allendorf, F. W. (2010). Estimation of census and effective population sizes: The increasing usefulness of DNA‐based approaches. Conservation Genetics, 11, 355–373. 10.1007/s10592-010-0050-7

[eva12731-bib-0050] Lynch, M. , Conery, J. , & Burger, R. (1995). Mutation accumulation and the extinction of small populations. American Naturalist, 146, 489–518. 10.1086/285812

[eva12731-bib-0051] Madsen, T. , Shine, R. , Olsson, M. , & Wittzell, H. (1999). Restoration of an inbred Adder population. Nature, 402, 34–35. 10.1038/46941

[eva12731-bib-0052] Madsen, T. , Ujvari, B. , & Olsson, M. (2004). Novel genes continue to enhance population growth of an inbred population of Adders (*Vipera berus*). Biological Conservation, 120, 145–147.

[eva12731-bib-0053] Marjoram, P. , & Tavare, S. (2006). Modern computational approaches for analysing molecular genetic variation data. Nature Reviews Genetics, 7, 759–770. 10.1038/nrg1961 16983372

[eva12731-bib-0054] McClusky, E. M. , Matthews, S. N. , Ligocki, I. Y. , Holding, M. L. , Lipps, G. J. , & Hetherington, T. E. (2018). The importance of historical land use in the maintenance of early successional habitat for a threatened rattlesnake. Global Ecology and Conservation, 13, e00370 10.1016/j.gecco.2017.e00370

[eva12731-bib-0055] Nachman, M. W. , & Crowell, S. L. (2000). Estimate of the mutation rate per nucleotide in humans. Genetics, 16, 297–304. 10.1093/genetics/156.1.297 PMC1461236 10978293

[eva12731-bib-0056] Palstra, F. P. , & Ruzzante, D. E. (2008). Genetic estimates of contemporary effective population size: What can they tell us about the importance of genetic stochasticity for wild population persistence? Molecular Ecology, 17, 3428–3447. 10.1111/j.1365-294X.2008.03842.x 19160474

[eva12731-bib-0057] Peery, M. Z. , Kirby, R. , Reid, B. N. , Stoelting, R. , Doucet‐Bëer, E. , Robinson, S. J. , … Palsbøll, P. J. (2012). Reliability of genetic bottleneck tests for detecting recent population declines. Molecular Ecology, 21, 3403–3418. 10.1111/j.1365-294X.2012.05635.x 22646281

[eva12731-bib-0058] Peig, J. , & Green, A. J. (2009). New perspectives for estimating body condition from mass/length data: The scaled mass index as an alternative method. Oikos, 118, 1883–1891. 10.1111/j.1600-0706.2009.17643.x

[eva12731-bib-0059] Peng, B. , & Kimmel, M. (2005). simuPOP: A forward‐time population genetics simulation environment. Bioinformatics, 21, 3686–3687. 10.1093/bioinformatics/bti584 16020469

[eva12731-bib-0060] Perrier, C. , Ferchaud, A.‐L. , Sirois, P. , Thibault, I. , & Bernatchez, L. (2017). Do genetic drift and accumulation of deleterious mutations preclude adaptation? Empirical investigation using RADseq in a northern lacustrine fish. Molecular Ecology, 26, 6317–6335. 10.1111/mec.14361 29024140

[eva12731-bib-0061] Peterson, B. K. , Weber, J. N. , Kay, E. H. , Fisher, H. S. , & Hoekstra, H. E. (2012). Double digest RADseq: An inexpensive method for de novo SNP discovery and genotyping in model and non‐model species. PLoS ONE, 7, e37135 10.1371/journal.pone.0037135 22675423 PMC3365034

[eva12731-bib-0062] Pielou, E. C. (1991). After the Ice Age: The Return of Life to Glaciated North America. Chicago, IL: University of Chicago Press.

[eva12731-bib-0063] Ralls, K. , Ballou, J. D. , Dudash, M. R. , Eldridge, M. D. B. , Fenster, C. B. , Lacy, R. C. , … Frankham, R. (2017). Call for a paradigm shift in the genetic management of fragmented populations. Conservation Letters, 11(2), e12412 10.1111/conl.12412.

[eva12731-bib-0064] Reed, D. H. , & Frankham, R. (2003). Correlation between fitness and genetic diversity. Conservation Biology, 17, 230–237. 10.1046/j.1523-1739.2003.01236.x

[eva12731-bib-0065] Rogers, R. L. , & Slatkin, M. (2017). Excess of genomic defects in a woolly mammoth on Wrangel Island. PLoS Genetics, 13, e1006601 10.1371/journal.pgen.1006601 28253255 PMC5333797

[eva12731-bib-0066] Rousset, F. (2008). Genepop’007: A complete re‐implementation of the genepop software for Windows and Linux. Molecular Ecology Resources, 8, 103–106. 10.1111/j.1471-8286.2007.01931.x 21585727

[eva12731-bib-0067] Salmona, J. , Salamolard, M. , Fouillot, D. , Ghestemme, T. , Larose, J. , Centon, J.‐F. , … Chikhi, L. (2012). Signature of a pre‐human population decline in the critically endangered reunion island endemic forest bird *Coracina newtoni* . PLoS ONE, 7, e43524 10.1371/journal.pone.0043524 22916272 PMC3423348

[eva12731-bib-0068] Schmidt, K. P. (1938). Herpetological evidence for the post‐glacial extension of the steppe in North America. Ecology, 19, 396–407.

[eva12731-bib-0069] Soltis, D. E. , Morris, A. B. , McLachlan, J. S. , Manos, P. , & Soltis, P. S. (2006). Comparative phylogeography of unglaciated eastern North America. Molecular Ecology, 15, 4261–4293. 10.1111/j.1365-294X.2006.03061.x 17107465

[eva12731-bib-0070] Sovic, M. G. , Fries, A. C. , & Gibbs, H. L. (2015). AftrRAD: A pipeline for accurate and efficient de novo assembly of RADseq data. Molecular Ecology Resources, 15, 1163–1171. 25641221 10.1111/1755-0998.12378

[eva12731-bib-0071] Sovic, M. G. , Fries, A. C. , & Gibbs, H. L. (2016). Origin of a cryptic lineage in a threatened reptile through isolation and historical hybridization. Heredity, 117, 358–366. 10.1038/hdy.2016.56 27460499 PMC5061922

[eva12731-bib-0072] Storz, J. F. , & Beaumont, M. (2002). Testing for genetic evidence of population expansion and contraction: An empirical analysis of microsatellite DNA variation using a hierarchical Bayesian method. Evolution, 56, 154–166. 11913661 10.1111/j.0014-3820.2002.tb00857.x

[eva12731-bib-0073] Szymanski, J. , Pollack, C. , Ragan, L. , Redmer, M. , Clemency, L. , Voorhies, K. , & JaKa, J . (2016). Species Status Assessment for the Eastern Massasauga Rattlesnake (Sistrurus catenatus). US Fish and Wildlife Service.

[eva12731-bib-0074] Tallmon, D. , Luikart, G. , & Waples, R. (2004). The alluring simplicity and complex reality of genetic rescue. Trends in Ecology and Evolution, 19, 489–496. 10.1016/j.tree.2004.07.003 16701312

[eva12731-bib-0075] Thomé, M. C. T. , & Carstens, B. C. (2016). Phylogeographic model selection leads to insight into the evolutionary history of Four‐eyed frogs. PNAS, 113, 8010–8017. 10.1073/pnas.1601064113 27432969 PMC4961127

[eva12731-bib-0076] Tucker, J. M. , Schwartz, M. K. , Truex, R. L. , Pilgrim, K. L. , & Allendorf, F. W. (2012). Historical and contemporary DNA indicate Fisher decline and isolation occurred prior to the European settlement of California. PLoS ONE, 7, e52803 10.1371/journal.pone.0052803 23300783 PMC3530519

[eva12731-bib-0077] US Fish and Wildlife Service (2016). Final Rule: Endangered and threatened wildlife and plants; Threatened species status for the eastern massasauga rattlesnake. Federal Register, 81, 67193–67214.

[eva12731-bib-0078] Wang, J. (2016). A comparison of single‐sample estimators of effective population sizes from genetic marker data. Molecular Ecology, 25, 4692–4711. 10.1111/mec.13725 27288989

[eva12731-bib-0079] Waples, R. S. (2016). Making sense of genetic estimates of effective population size. Molecular Ecology, 25, 4689–4691. 10.1111/mec.13814 27671356

[eva12731-bib-0080] Waples, R. S. , Antao, T. , & Luikart, G. (2014). Effects of overlapping generations on linkage disequilibrium estimates of effective population size. Genetics, 197, 769–780. 10.1534/genetics.114.164822 24717176 PMC4063931

[eva12731-bib-0081] Waples, R. S. , & Do, C. (2008). LdNe: A program for estimating effective population size from data on linkage disequilibrium. Molecular Ecology Resources, 8, 753–756. 21585883 10.1111/j.1755-0998.2007.02061.x

[eva12731-bib-0082] Waples, R. S. , & Do, C. (2010). Linkage disequilibrium estimates of contemporary Ne using highly variable genetic markers: A largely untapped resource for applied conservation and evolution. Evolutionary Applications, 3, 244–262. 25567922 10.1111/j.1752-4571.2009.00104.x PMC3352464

[eva12731-bib-0083] Waples, R. K. , Larson, W. A. , & Waples, R. S. (2016). Estimating contemporary effective population size in non‐model species using linkage disequilibrium across thousands of loci. Heredity, 117, 233–240. 10.1038/hdy.2016.60 27553452 PMC5026758

[eva12731-bib-0084] Whitlock, M. C. , & Lotterhos, K. E. (2015). Reliable detection of loci responsible for local adaptation: Inference of a neutral model through trimming the distribution of Fst. American Naturalist, 186, S24–S36. 10.1086/682949 26656214

[eva12731-bib-0085] Wood, J. L. A. , Yates, M. C. , & Fraser, D. J. (2016). Are heritability and selection related to population size in nature? Meta‐analysis and conservation implications. Evolutionary Applications, 9, 640–657. 10.1111/eva.12375 27247616 PMC4869407

[eva12731-bib-0086] Zhu, L. F. , Hu, Y. B. , Qi, D. W. , Wu, H. , Zhan, X. , Zhang, Z. , … Wei, F. . (2013). Genetic consequences of historical anthropogenic and ecological events on giant pandas. Ecology, 94, 2346–2357. 10.1890/12-1451.1 24358719

