# Supplementary material for: Genetic signatures of small effective population sizes and demographic declines in an endangered rattlesnake, Sistrurus catenatus
Source: Evol Appl. 2019 Jan 28;12(4):664–78. doi: 10.1111/eva.12731 (PMC6439488; doi:10.1111/eva.12731)
Supplement: Supplementary file 1 [file EVA-12-664-s001.docx]

**Supplemental Material**

Table S1a: Pairwise Fst values based on RADseq data

|  | SSSP | EHSP | SPVY | PRDF | KLDR | WLRD | GRL-1 | GRL-2 | GRL-3 | GLAD | VNGO | JENN | BERG | CCRO | BPNP | BEAU | KBPP |
| --- | --- | --- | --- | --- | --- | --- | --- | --- | --- | --- | --- | --- | --- | --- | --- | --- | --- |
| SSSP | 0.000 |  |  |  |  |  |  |  |  |  |  |  |  |  |  |  |  |
| EHSP | 0.088 | 0.000 |  |  |  |  |  |  |  |  |  |  |  |  |  |  |  |
| SPVY | 0.392 | 0.339 | 0.000 |  |  |  |  |  |  |  |  |  |  |  |  |  |  |
| PRDF | 0.398 | 0.354 | 0.350 | 0.000 |  |  |  |  |  |  |  |  |  |  |  |  |  |
| KLDR | 0.248 | 0.205 | 0.191 | 0.215 | 0.000 |  |  |  |  |  |  |  |  |  |  |  |  |
| WLRD | 0.287 | 0.241 | 0.196 | 0.276 | 0.127 | 0.000 |  |  |  |  |  |  |  |  |  |  |  |
| GRL-1 | 0.334 | 0.282 | 0.264 | 0.288 | 0.153 | 0.181 | 0.000 |  |  |  |  |  |  |  |  |  |  |
| GRL-2 | 0.418 | 0.362 | 0.361 | 0.363 | 0.188 | 0.237 | 0.228 | 0.000 |  |  |  |  |  |  |  |  |  |
| GRL-3 | 0.336 | 0.273 | 0.272 | 0.322 | 0.154 | 0.176 | 0.162 | 0.225 | 0.000 |  |  |  |  |  |  |  |  |
| GLAD | 0.427 | 0.366 | 0.376 | 0.361 | 0.239 | 0.289 | 0.303 | 0.363 | 0.314 | 0.000 |  |  |  |  |  |  |  |
| VNGO | 0.373 | 0.303 | 0.352 | 0.344 | 0.221 | 0.253 | 0.266 | 0.360 | 0.257 | 0.365 | 0.000 |  |  |  |  |  |  |
| JENN | 0.405 | 0.325 | 0.355 | 0.372 | 0.223 | 0.269 | 0.292 | 0.357 | 0.283 | 0.406 | 0.314 | 0.000 |  |  |  |  |  |
| BERG | 0.500 | 0.444 | 0.454 | 0.462 | 0.283 | 0.369 | 0.366 | 0.449 | 0.402 | 0.512 | 0.490 | 0.457 | 0.000 |  |  |  |  |
| CCRO | 0.494 | 0.454 | 0.479 | 0.461 | 0.313 | 0.405 | 0.396 | 0.473 | 0.404 | 0.491 | 0.475 | 0.465 | 0.443 | 0.000 |  |  |  |
| BPNP | 0.277 | 0.226 | 0.247 | 0.249 | 0.125 | 0.157 | 0.168 | 0.223 | 0.161 | 0.267 | 0.230 | 0.262 | 0.304 | 0.294 | 0.000 |  |  |
| BEAU | 0.339 | 0.305 | 0.289 | 0.319 | 0.176 | 0.225 | 0.261 | 0.287 | 0.229 | 0.338 | 0.296 | 0.310 | 0.326 | 0.314 | 0.151 | 0.000 |  |
| KBPP | 0.303 | 0.260 | 0.272 | 0.304 | 0.155 | 0.210 | 0.242 | 0.275 | 0.228 | 0.346 | 0.259 | 0.310 | 0.363 | 0.370 | 0.132 | 0.155 | 0.000 |

|  | SSSP | EHSP | SPVY | PRDF | KLDR | WLRD | GRL-1 | GRL-2 | GRL-3 | GLAD | VNGO | JENN | BERG | CCRO | BPNP | BEAU | KBPP |
| --- | --- | --- | --- | --- | --- | --- | --- | --- | --- | --- | --- | --- | --- | --- | --- | --- | --- |
| SSSP | 0.000 |  |  |  |  |  |  |  |  |  |  |  |  |  |  |  |  |
| EHSP | 0.088 | 0.000 |  |  |  |  |  |  |  |  |  |  |  |  |  |  |  |
| SPVY | 0.392 | 0.339 | 0.000 |  |  |  |  |  |  |  |  |  |  |  |  |  |  |
| PRDF | 0.398 | 0.354 | 0.350 | 0.000 |  |  |  |  |  |  |  |  |  |  |  |  |  |
| KLDR | 0.248 | 0.205 | 0.191 | 0.215 | 0.000 |  |  |  |  |  |  |  |  |  |  |  |  |
| WLRD | 0.287 | 0.241 | 0.196 | 0.276 | 0.127 | 0.000 |  |  |  |  |  |  |  |  |  |  |  |
| GRL-1 | 0.334 | 0.282 | 0.264 | 0.288 | 0.153 | 0.181 | 0.000 |  |  |  |  |  |  |  |  |  |  |
| GRL-2 | 0.418 | 0.362 | 0.361 | 0.363 | 0.188 | 0.237 | 0.228 | 0.000 |  |  |  |  |  |  |  |  |  |
| GRL-3 | 0.336 | 0.273 | 0.272 | 0.322 | 0.154 | 0.176 | 0.162 | 0.225 | 0.000 |  |  |  |  |  |  |  |  |
| GLAD | 0.427 | 0.366 | 0.376 | 0.361 | 0.239 | 0.289 | 0.303 | 0.363 | 0.314 | 0.000 |  |  |  |  |  |  |  |
| VNGO | 0.373 | 0.303 | 0.352 | 0.344 | 0.221 | 0.253 | 0.266 | 0.360 | 0.257 | 0.365 | 0.000 |  |  |  |  |  |  |
| JENN | 0.405 | 0.325 | 0.355 | 0.372 | 0.223 | 0.269 | 0.292 | 0.357 | 0.283 | 0.406 | 0.314 | 0.000 |  |  |  |  |  |
| BERG | 0.500 | 0.444 | 0.454 | 0.462 | 0.283 | 0.369 | 0.366 | 0.449 | 0.402 | 0.512 | 0.490 | 0.457 | 0.000 |  |  |  |  |
| CCRO | 0.494 | 0.454 | 0.479 | 0.461 | 0.313 | 0.405 | 0.396 | 0.473 | 0.404 | 0.491 | 0.475 | 0.465 | 0.443 | 0.000 |  |  |  |
| BPNP | 0.277 | 0.226 | 0.247 | 0.249 | 0.125 | 0.157 | 0.168 | 0.223 | 0.161 | 0.267 | 0.230 | 0.262 | 0.304 | 0.294 | 0.000 |  |  |
| BEAU | 0.339 | 0.305 | 0.289 | 0.319 | 0.176 | 0.225 | 0.261 | 0.287 | 0.229 | 0.338 | 0.296 | 0.310 | 0.326 | 0.314 | 0.151 | 0.000 |  |
| KBPP | 0.303 | 0.260 | 0.272 | 0.304 | 0.155 | 0.210 | 0.242 | 0.275 | 0.228 | 0.346 | 0.259 | 0.310 | 0.363 | 0.370 | 0.132 | 0.155 | 0.000 |

Table S1b: Pairwise Fst values based on microsatellites

Table S2: Ne estimates from LDNe (RAD), LDNe (Msat), Colony (RAD), and Colony (Msat).

Values in parentheses represent 95% confidence intervals based on jackknife and bootstrap resampling for the LDNe and Colony analyses, respectively. NA means an estimate could not be obtained or that it was unreasonable (see Results); ND means that no data was available.

|  | LDNe |  | Colony |  |
| --- | --- | --- | --- | --- |
| Population | RAD | Micro | RAD | Micro |
| BEAU | 22 (18-27) | 48 (22-Inf) | 62 (38-136) | 35 (19-82) |
| BERG | 9 (6-13) | 7 (5-10) | 23 (11-56) | 19 (10-39) |
| BPNP | 48 (39-63) | NA | 140 (75-370) | 95 (50-318) |
| CCRO | 13 (10-16) | 22 (19-26) | 21 (12-43) | 38 (25-61) |
| EHSP | NA | 23 (14-46) | NA | 61 (26-Inf) |
| SSSP | 22 (9-Inf) | 20 (14-34) | NA | 34 (19-78) |
| GLAD | 6 (5-194) | 51 (6-Inf) | NA | NA |
| GRL-3 | 7 (6-8) | 9 (7-12) | 25 (14-50) | 35 (19-77) |
| JENN | 2 (2-3) | 64 (13-Inf) | 20 (9-69) | 48 (18-Inf) |
| KBPP | 10 (9-12) | 14 (10-19) | 26 (13-57) | 32 (18-67) |
| KLDR | 44 (37-53) | 33 (30-35) | 74 (44-148) | 74 (54-106) |
| GRL-2 | 9 (7-12) | 4 (3-7) | 18 (10-42) | 20 (10-41) |
| PRDF | 24 (18-32) | 18 (13-27) | 32 (18-64) | 30 (17-59) |
| GRL-1 | 3 (3-3) | 3 (3-3) | 15 (7-34) | 14 (7-33) |
| SPVY | 8 (3-19) | ND | 8 (3-30) | ND |
| VNGO | NA | 13 (3-Inf) | NA | 84 (17-Inf) |
| WLRD | 33 (20-77) | 17 (12-27) | NA | 42 (20-131) |

Table S3. AIC model selection results from *fastsimcoal* analyses based on RADseq data for each of 17 *S. catenatus* populations. A model of constant population size (null) was compared to a model that incorporates population size change (bottleneck). Bold text indicates the model to which the greatest relative weight was given for each population.

|  | # Parameters | Ln Likelihood | AIC | Akaike weight |
| --- | --- | --- | --- | --- |
| SSSP |  |  |  |  |
| *Null* | 1 | -1152.11 | 5307.64 | 9.11e^-7^ |
| ***Bottleneck*** | **3** | **-1145.20** | **5279.83** | **1** |
| EHSP |  |  |  |  |
| *Null* | 1 | -1258.25 | 5796.46 | 3.17e^-5^ |
| ***Bottleneck*** | **3** | **-1252.88** | **5775.74** | **1** |
| SPVY |  |  |  |  |
| *Null* | 1 | -886.03 | 4082.33 | 1.30e^-11^ |
| ***Bottleneck*** | **3** | **-874.28** | **4032.20** | **1** |
| PRDF |  |  |  |  |
| *Null* | 1 | -964.96 | 4445.81 | 1.19e^-26^ |
| ***Bottleneck*** | **3** | **-938.17** | **4326.43** | **1** |
| KLDR |  |  |  |  |
| *Null* | 1 | -1454.61 | 6700.70 | 3.90e^-12^ |
| ***Bottleneck*** | **3** | **-1442.33** | **6648.16** | **1** |
| WLRD |  |  |  |  |
| *Null* | 1 | -1421.94 | 6550.28 | 1.02e^-11^ |
| ***Bottleneck*** | **3** | **-1410.08** | **6499.65** | **1** |
| ROME |  |  |  |  |
| *Null* | 1 | -1018.20 | 4690.99 | 3.34e^-23^ |
| ***Bottleneck*** | **3** | **-994.86** | **4587.48** | **1** |
| MOSQ |  |  |  |  |
| *Null* | 1 | -1020.00 | 4699.28 | 1.38e^-16^ |
| ***Bottleneck*** | **3** | **-1003.27** | **4626.24** | **1** |
| GYSH |  |  |  |  |
| *Null* | 1 | -1211.96 | 5583.29 | 1.83e^-21^ |
| ***Bottleneck*** | **3** | **-1190.36** | **5487.79** | **1** |
| GLAD |  |  |  |  |
| *Null* | 1 | -811.29 | 3738.11 | 3.60e^-5^ |
| ***Bottleneck*** | **3** | **-805.98** | **3717.65** | **1** |
| VNGO |  |  |  |  |
| *Null* | 1 | -939.57 | 4328.88 | 2.33e^-10^ |
| ***Bottleneck*** | **3** | **-929.07** | **4284.53** | **1** |
| JENN |  |  |  |  |
| *Null* | 1 | -981.51 | 4522.03 | 1.13e^-13^ |
| ***Bottleneck*** | **3** | **-967.70** | **4462.41** | **1** |
| BERG |  |  |  |  |
| *Null* | 1 | -803.77 | 3703.48 | 1.71e^-13^ |
| ***Bottleneck*** | **3** | **-790.13** | **3644.69** | **1** |
| CCRO |  |  |  |  |
| *Null* | 1 | -921.07 | 4243.67 | 1.62e^-13^ |
| ***Bottleneck*** | **3** | **-907.41** | **4184.77** | **1** |
| BPNP |  |  |  |  |
| *Null* | 1 | -1322.26 | 6091.24 | 8.08e^-17^ |
| ***Bottleneck*** | **3** | **-1305.30** | **6017.13** | **1** |
| BEAU |  |  |  |  |
| *Null* | 1 | -1289.97 | 5942.54 | 4.27e^-19^ |
| ***Bottleneck*** | **3** | **-1270.73** | **5857.95** | **1** |
| KBPP |  |  |  |  |
| *Null* | 1 | -1348.74 | 6213.17 | 1.57e^-14^ |
| ***Bottleneck*** | **3** | **-1334.07** | **6149.61** | **1** |

Table S4: Parameter estimates for the number of generations and years in the past when the population size change occurred (TBot), and the long-term coalescent-based current (Nc) and ancestral (Na) genetically effective population sizes. Estimates were generated under the optimal (bottleneck) model in fastsimcoal (RADseq).

| RADseq | | | | | | |
| --- | --- | --- | --- | --- | --- | --- |
| Population | TBot (yrs) | CI (gens) | Na | CI | Nc | CI |
| BEAU | 578 (1173) | 10-5447 | 32658 | 30189-54908 | 2028 | 30-6113 |
| BERG | 3 (6) | 34-362 | 21073 | 18540-47101 | 11 | 11-682 |
| BPNP | 6207 (12600) | 2265-10941 | 56684 | 39361-73324 | 8879 | 6327-1243 |
| CCRO | 4076 (8274) | 997-7242 | 40552 | 31543-6664 | 3670 | 1200-4878 |
| EHSP | 811 (1646) | 13-9874 | 35999 | 34383-48983 | 6302 | 122-20137 |
| SSSP | 71 (144) | 4-248 | 39500 | 35690-49224 | 320 | 18-1114 |
| GLAD | 12 (24) | 8-338 | 31751 | 22991-48635 | 23 | 11-361 |
| GRL-3 | 6 (12) | 3-124 | 32244 | 29460-37080 | 27 | 11-717 |
| JENN | 4 (8) | 3-428 | 26982 | 23379-35682 | 15 | 14-878 |
| KBPP | 1504 (3053) | 5-543 | 34688 | 29538-48542 | 4942 | 37-8426 |
| KLDR | 7841 (15917) | 2693-14448 | 49292 | 36935-64760 | 12583 | 9930-16578 |
| GRL-2 | 1183 (2401) | 5-2695 | 47963 | 28606-52463 | 1408 | 12-3383 |
| PRDF | 960 (1949) | 10-2868 | 56215 | 44189-71397 | 1045 | 11-2490 |
| GRL-1 | 95 (193) | 4-1250 | 34492 | 29321-52818 | 255 | 12-2031 |
| SPVY | 12 (24) | 9-1369 | 48407 | 35904-55515 | 16 | 14-1805 |
| VNGO | 6 (12) | 4-362 | 41068 | 29661-53920 | 10 | 11-588 |
| WLRD | 5 (10) | 7-5418 | 36775 | 35893-50194 | 28 | 28-10719 |
